# Impact of cartilage graft size on success of tympanoplasty^☆^

**DOI:** 10.1016/j.bjorl.2016.06.005

**Published:** 2016-07-12

**Authors:** Waleed Abdelhameed, Ibrahim Rezk, Alhussein Awad

**Affiliations:** Sohag University, Department of Otorhinolaryngology, Sohag, Egypt

**Keywords:** Prospective study of cartilage graft, Size of perforation, Tympanoplasty, Estudo prospectivo de enxerto de cartilagem, Tamanho da perfuração, Timpanoplastia

## Abstract

**Introduction:**

In the last decade, there has been an increasing use of cartilage grafts in the primary repair of tympanic membrane perforations. The major advantages of cartilage are its stiffness and its very low metabolic requirements, which make it particularly suitable for difficult conditions, such as subtotal perforations, adhesive otitis and reoperation.

**Objective:**

To analyze the impact of different perforation sizes requiring different sizes of cartilage on the anatomical and functional outcome after tympanoplasty.

**Methods:**

Through this prospective non-controlled, non-randomized study, 50 patients underwent cartilage type 1 tympanoplasty (20 females and 30 males), with a mean age of 19.3 ± 9.8 years. According to size of perforation, patients were subdivided into three groups, Group I had perforation >50% of tympanic membrane area, in Group II patients the perforations were 25–50% of tympanic membrane area, and in Group III the perforations were ≤25% of tympanic membrane. All patients had pre and postoperative Pure Tone Average and Air Bone Gap frequencies (0.5, 1, 2, 4 kHz). All patients were followed up at least 12 months after operation.

**Results:**

The anatomical success rate among all patients was 92%, all groups showed statistical significant improvement between pre and postoperative air bone gap, no significant correlation between size of cartilage graft and degree of air bone gap improvement was noticed among the three groups.

**Conclusion:**

Size of a cartilage graft has no impact on degree of hearing improvement or anatomical success rate after tympanoplasty.

## Resumo

**Introdução:**

Na última década, tem havido um interesse crescente na utilização de enxertos de cartilagem como opção para o reparo de perfurações primárias de membrana timpânica. As principais vantagens da cartilagem são a sua rigidez e metabolismo braditrófico, o que a torna particularmente adequada para condições difíceis, tais como perfurações subtotais, otite adesiva e reoperações.

**Objetivo:**

Analisar o impacto de diferentes tamanhos de perfuração, portanto, diferentes tamanhos de cartilagem sobre o desfecho anatômico e funcional da timpanoplastia.

**Método:**

Através deste estudo prospectivo, não controlado, não randomizado, 50 pacientes foram submetidos a timpanoplastia de cartilagem tipo 1 (20 mulheres e 30 homens), com idade média de 19,3 ± 9,8 anos. De acordo com o tamanho da perfuração, os pacientes foram subdivididos em três grupos, Grupo I com perfuração > 50% da área da membrana timpânica, Grupo II com perfuração de 25%-50% da área da membrana timpânica, Grupo III com perfuração ≤ 25% da membrana timpânica. Todos apresentavam Audiometria tonal pura pré e pós-operatório - gap Aéreo-Ósseo para frequências testadas (0,5, 1, 2, 4 kHz). Todos os pacientes foram acompanhados por pelo menos 12 meses após a operação.

**Resultados:**

A taxa de sucesso anatômico entre todos os pacientes foi de 92%, todos os grupos apresentaram melhoria estatisticamente significante entre pré e pós-operatório nos três grupos, não houve correlação significante entre o tamanho do enxerto de cartilagem e observou-se algum grau de melhoria do gap nos 3 grupos.

**Conclusão:**

O tamanho do enxerto de cartilagem não tem impacto sobre o grau de melhoria da audição ou na taxa de sucesso anatômico após timpanoplastia.

## Introduction

The fascia temporalis is the most commonly used graft in primary tympanoplasty, with success rate between 93% and 97%, especially in well-aerated middle ears.^1^, ^2^

In the last decade, however there has been an increasing interest in using cartilage grafts as the primary alternative.^3^ The major advantages of cartilage are its stiffness and bradytrophic metabolism, which make it particularly suitable for difficult conditions, such as subtotal perforations, adhesive otitis and reoperation.^4^

This material is also characterized by its resistance to resorption, retraction and negative pressure in the middle ear, its connection to the surrounding tissue and its elasticity suitable for sound transmission.^5^, ^6^, ^7^, ^8^

The size of the perforation is thought to play a role in determining the success of myringoplasty. For some authors, large perforations are often associated with lower success rate possibly due to surgical difficulties. Obviously, these perforations require more graft material and are often associated with poorer condition of the remaining tympanic membrane.^9^, ^10^

However, for other authors, the preoperative perforation size does not correlate with the success rate of the operation.^11^, ^12^

Many studies discussed the effect of perforation size on the success of tympanoplasty. Our literature search suggests no studies looked for the same effect on using cartilage as a graft.

## Objective

To analyze impact of different perforation sizes hence different sizes of cartilage graft on the anatomical and functional outcome of tympanoplasty.

## Patients and methods

### Subjects

A prospective non-controlled, non-randomized study was conducted at ENT department, Sohag University Hospital. The protocol of investigation had been approved by our Ethics of Research Committee (Number 12/2013). The investigators had obtained written consent from each participant or their guardians. It included fifty patients undergoing type 1 cartilage tympanoplasty, from August 2013 to July 2014.

**Inclusion criteria:** patients who had chronic otitis media, and need type 1 tympanoplasty, with dry ear, and normal middle ear mucosa, at least 3 months preoperatively.

**Exclusion criteria:** patients who had previous ear surgery, or requiring concomitant mastoidectomy, those with atalectatic tympanic membranes or with cholesteatoma.

**Audiometric tests:** Include preoperative and postoperative Pure Tone Average (PTA)–Air Bone Gap (ABG) for tested frequencies (0.5, 1, 2, 4 kHz), done at our Audiology unit.

### Surgery

**Figure 1:**
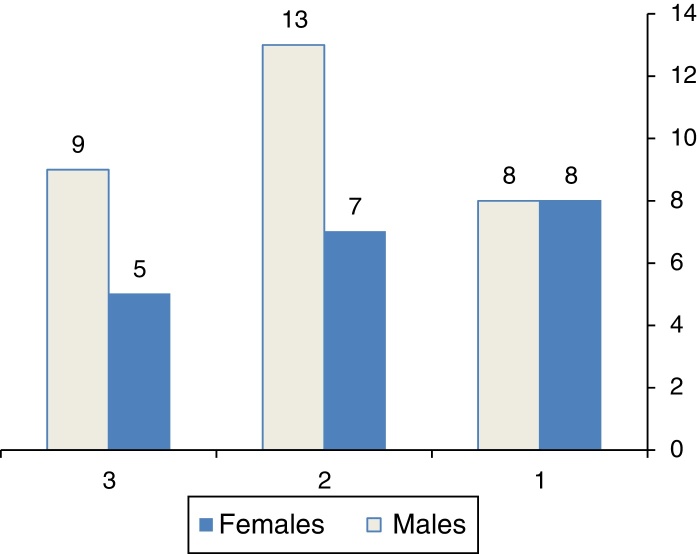
Sex of patients among three groups, G1 (perforation size > 50% of TM area), G2 (perforation size between 25% and 50% of TM area), G3 (perforation size ≤ 25% of TM area).

Size of perforation was calculated at time of operation by applying a piece of Silastic to fit snugly with perforation, then taken out and measured at longitudinal and transverse diameters. With considering that adult tympanic membrane size to be 10 mm × 9 mm, patients were subdivided into three groups according to perforation size, Group I with perforation size more than 45 mm^2^ (>50% of tympanic membrane area), Group II with perforation size between 23 mm^2^ and 45 mm^2^ (25–50% of tympanic membrane area), Group III perforation was ≤25% of tympanic membrane area (23 mm^2^), this is shown in Fig. 1 and Table 1.

**Table 1:** Demographic characters of patients among three groups.

Sex	Mean age	Mean perforation size	Mean ABG diff.
*First group (>50% of TM area)*			
Males 8	19.6 ± 6.07 y	51.8 ± 10.41 mm^2^	9.53 ± 5.6 dB
Females 8			

*Second group (25–50% of TM area)*			
Males 13	20.2 ± 11.65 y	32.15 ± 5.64 mm^2^	10.76 ± 6.59 dB
Females 7			

*Third group (≤25% of TM area)*			
Males 9	17.35 ± 10.34 y	17.85 ± 2.34 mm^2^	10.6 ± 4.27 dB
Females 5			

The patients were operated upon by the first two senior authors, all procedures were performed under general anesthesia, using a post auricular approach, and grafts from tragus were harvested. The cartilage thickness did not exceed 0.5 mm.

The cartilage perichondrium graft was prepared by dissecting perichondrium off one side only, keeping the attachment of other side, the graft was placed by underlay technique with elevated perichondrium draping on to posterior canal wall.

### Outcome measures

We measured anatomical and functional success, the first was defined as full healing of grafted tympanic membrane for at least 12 months postoperatively.

Patients repeated the audiogram at 6.9 months postoperatively; hearing outcomes were calculated from the most recent audiogram, by comparing pre and postoperative Pure Tone Average–Air Bone Gap (PTA–ABG), hearing gain was defined as the difference between both.

### Statistical analysis

Data were analyzed using Statistical Package for the Social Sciences version 18.0 (SPSS Inc, Chicago, IL, USA). We used paired *t*-test for studying change of PTA–ABG, and partial correlation to study the relation between size of cartilage graft and degree of improvement in ABG. According to numbers of 16, 20 and 14 patients in the three studied groups, the power of study was 0.95033 (95%).

## Results

Fifty patients underwent cartilage tympanoplasty (20 females and 30 males), their age range was (9–65 years) with a mean of 19.3 ± 9.8 years. Thirty seven patients had unilateral perforation, while 13 had bilateral perforations. Hearing loss was the main symptom in (82%), recurrent otorrhea in (75%), and tinnitus in (9%) of patients. Right side was the operated side in 21 patients, and left side in the remaining group. Patients’ data were shown in Table 1. All patients were followed up at least 12 months after operation with range (12–20 months), with no recorded lost follow up among them.

The anatomical success rate defined as graft take after 12 months of follow up, among all patients was 92%, 4 patients underwent revision surgery 10–12 months postoperatively. Two of them were in first group, and one patient in each remaining groups, no statistical difference was noted among the three groups regarding failure percentage.

**Figure 2:**
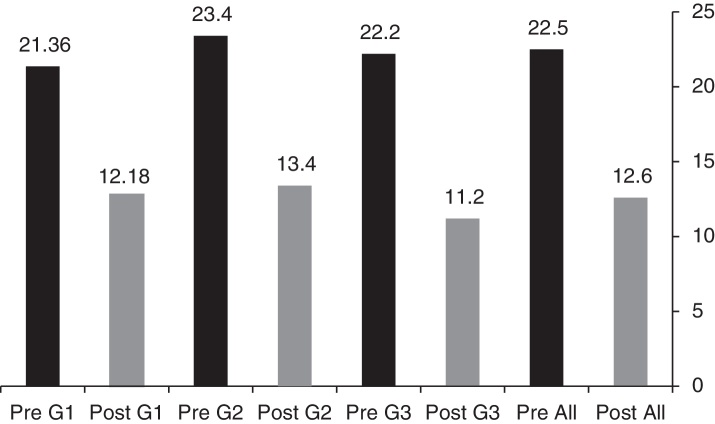
ABG mean in dBs pre and postoperatively of the three groups, and for the total number of patients.

Regarding functional outcome, all groups showed statistical significant improvement between pre and postoperative (PTA–ABG), the mean ABG preoperatively in all patients was 22.5 dB, that of postoperative was 12.6 dB (*p* = 0.000), for each group the ABG pre and postoperatively is shown in Fig. 2. The mean of ABG difference (Pre-Postop) was also demonstrated in Table 1.

On searching for the correlation between the sizes of perforation, hence size of cartilage graft, and degree of improvement in ABG, no significant correlation was noticed, among the three groups as outlined in Table 2.

**Table 2:** The partial correlation between size of perforation and ABG improvement postoperatively between three groups.

	Pair (I)	Pair (II)	Pair (III)
*Pair (I)*			
Correlation *r*	–	0.018	−0.066
Significance 2 tailed		0.952	0.0847
		*p*-Value NS	*p*-Value NS

*Pair (II)*			
Correlation *r*	0.018		0.023
Significance 2 tailed	0.952	–	0.946
	*p*-Value NS		*p*-Value NS

*Pair (III)*			
Correlation *r*	−0.066	0.023	–
Significance 2 tailed	0.0847	0.946	
	*p*-Value NS	*p*-Value NS	

Each pair represents mean of size of perforation of that group with mean of ABG improvement in same group; *r*, correlation coefficient; *p*-value between groups >0.05 NS (non significant).

## Discussion

A more rigid, and more resorption- and retraction-resistant graft material, may provide better success rate. Therefore, cartilage graft materials are preferred for large perforations.^13^ Kazikdas et al. found 95.7% graft success rate for palisade cartilage graft, compared with 75% for temporalis fascia grafts.^14^

The anatomical success rate in our patients was 92%, this is comparable with rates mentioned in literature for cartilage tympanoplasty, 93% in study of Yurttas et al. and 92.3% in study by Onal et al.^13^, ^15^

Previous studies have assessed the relationship between the size of tympanic membrane perforations and hearing loss, with conflicting data and without proper methodology.^16^

In the literature, Pannu et al. reported different results, demonstrating an increase in hearing loss with increasing sizes of tympanic perforation in 100 patients.^17^ Also, Ibekwe et al. analyzed 67 patients with a total of 77 perforations, they concluded that the larger the tympanic membrane perforation, the greater the loss in sound perception.^18^

On the other hand, Ribeiro et al. found no significant relationship between the size of tympanic perforations and hearing loss in the four analyzed frequencies 0.5, 1, 2, 4 kHz.^16^ We searched for the link between cartilage graft size and change of ABG after tympanoplasty, No impact of size of cartilage graft on degree of ABG improvement was revealed.

If a larger cartilage plate is used for reconstruction, a smaller thickness of transplant is necessary. For optimal acoustic transfer behavior, the cartilage should be cut as thinly as possible,^19^ so we used cartilage graft with thickness not more than 0.5 mm.

Gerber et al. mentioned that replacing a large portion of tympanic membrane with cartilage would add stiffness and/or mass that would affect individual frequencies, but not significantly impact averaged audiometric data such as air bone gap.^20^ Our work proved that, as significant improvement of ABG was achieved among the three groups postoperatively.

Our patients with big sized perforation showed least improvement of ABG compared with other groups. This may be explained according to the hypothesis that increasing size of perforation will increase the degree of hearing loss. But their significant improvement of ABG postoperatively, proves also that cartilage size has no impact on averaged audiometric data such as ABG.

On studying the correlation between size of graft and degree of improvement of ABG, no significant correlation was found. This is coinciding with hypothesis of Ribeiro et al. who did not find any correlation between size of perforation and degree of hearing loss among four tested frequencies.^16^

## Conclusion

Cartilage tympanoplasty has high anatomical success rate (92%).

Size of cartilage graft has no impact on degree of hearing improvement or anatomical success rate after tympanoplasty.

## Limitations

To make our results more generalized, we need to investigate higher number of patients. This is not feasible, as cartilage is not the sole grafting material used at our department.

## Conflicts of interest

The authors declare no conflicts of interest.
